# Disrupting the *mtr*-operon in *Methanosarcina acetivorans* enables methyl-reducing methanogenesis with hydrogen and serine as the alternative electron donors

**DOI:** 10.1093/femsle/fnag061

**Published:** 2026-05-22

**Authors:** Jichen Bao, Tejas Somvanshi, Yufang Tian, Silvan Scheller

**Affiliations:** Department of Bioproducts and Biosystems, School of Chemical Engineering, Aalto University, Kemistintie 1, 02150 Espoo, Finland; Department of Bioproducts and Biosystems, School of Chemical Engineering, Aalto University, Kemistintie 1, 02150 Espoo, Finland; Department of Bioproducts and Biosystems, School of Chemical Engineering, Aalto University, Kemistintie 1, 02150 Espoo, Finland; Department of Bioproducts and Biosystems, School of Chemical Engineering, Aalto University, Kemistintie 1, 02150 Espoo, Finland

**Keywords:** methanosarcina acetivoransm, methyl-reducing methanogenesish, hydrogens, serine, hydrogenases

## Abstract

*Methanosarcina acetivorans* is a model methanogen because of its metabolic versatility and genetic tractability. This microbe is not known to natively utilize hydrogen as a catabolic electron donor, despite its genome encoding hydrogenases, and the fact that this microbe can be used to heterologously express functional hydrogenases. Its native hydrogenases are expressed at a very low level and are suggested to have a role in recycling hydrogen that is produced as a byproduct of nitrogen fixation. To explore whether hydrogen can act as a catabolic electron donor, we utilized a previously constructed strain (str. JB-MF) that has the operon encoding for the methyl-H_4_MPT:CoM methyltransferase (Mtr) disrupted which makes growth dependent on oxidation of electron donors other than methanol. We showed that this *M. acetivorans* strain can grow by methanol reduction to methane with hydrogen as the sole electron donor. The strain was then used to test the putative electron donors for methyl-reducing methanogenesis: hydrogen, serine, and ethanol. Hydrogen and serine were identified to act as catabolic electron donors for methyl-reducing methanogenesis, which demonstrates the expanded metabolic versatility of *M. acetivorans* JB-MF. The methanophenazine-reducing hydrogenase (Vht) and the F_420_-reducing hydrogenase (Frh) were determined to be involved in hydrogen metabolism, because deletion of either made *M. acetivorans* JB-MF incapable of growing using H_2_-dependent methyl-reduction. We demonstrate that strain JB-MF is a suitable chassis to screen alternative electron donors for methanogenesis and that this strain may be used for targeted directed evolution of oxidoreductases.

## Teaser

This study shows that the metabolic flexibility of *Methanosarcina acetivorans* extends to hydrogen-dependent and serine-dependent methyl-reducing methanogenesis.

## Introduction

**Figure 1:**
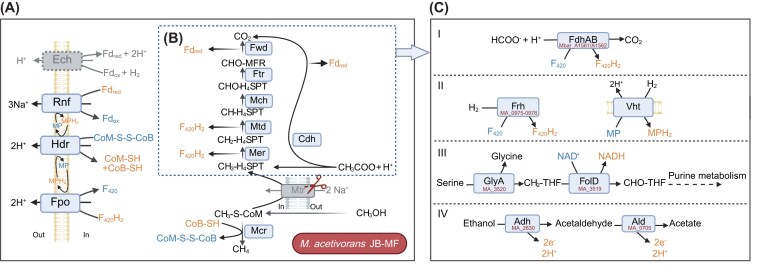
Overview of methyl-reducing methanogenesis pathways in *Methanosarcina acetivorans* JB-MF. (A) The disruption of the *mtr* operon restricts growth to methyl-reducing methanogenesis. The heterodisulfide formed needs to be reduced to keep methanogenesis going. *Methanosarcina acetivorans* relies on H_2_-independent electron transport chain because it lacks the energy-converting hydrogenase (Ech). The heterodisulfide is the ultimate electron acceptor and reduced cofactors Fd_red_ and F_420_H_2_ are used to shuttle electrons. (B) Native capability of *M. acetivorans* to generate Fd_red_ and F_420_H_2_ using acetate when Mtr is not expressed (Eq 3). (C) Alternative electron donors that *M. acetivorans* JB-MF can hypothetically oxidize to provide electrons for methyl-reduction are (I) formate—tested in previous study (Bao et al. 2025), and the following were tested in this study- (II) hydrogen, (III) serine, and (IV) ethanol. Enzymes: carbon monoxide dehydrogenase/acetyl-CoA synthase complex (CODH/ACS), methyl-H_4_MPT:CoM methyltransferase (Mtr), F_420_H_2_ dehydrogenase (Fpo), heterodisulfide reductase (Hdr), *Rhodobacter* nitrogen fixation complex (Rnf), formate dehydrogenase (Fdh), F_420_-reducing hydrogenase (Frh), methanophenazine-reducing hydrogenase (Vht), energy-converting hydrogenases (Ech), bifunctional N^5^,N^10^-methylene-H_4_F dehydrogenase/N^5^,N^10^-methenyl-H_4_F cyclohydrolase (FolD), serine:H_4_F hydroxymethyltransferase (GlyA), alcohol dehydrogenase (Adh), aldehyde dehydrogenase (Ald), methanol methyltransferase (Mta), and methyl-CoM reductase (Mcr). Note: *Methanosarcina* spp. use tetrahydrosarcinapterin, which is an analogue of H_4_MPT. Created with Biorender.com.

Methanogens play a crucial role in the global carbon cycle by producing ca. 1 Gt of methane annually. All methanogens in nature rely on methane formation for their energy metabolism (Thauer et al. 2008, Garcia et al. 2022). Their unifying feature is the reduction of a methyl group to methane via the key enzyme methyl-coenzyme M (CoM) reductase (Mcr) which uses electrons from coenzyme B to form CoM-S-S-CoB. A key enzyme in the energy metabolism of select methanogens is the membrane-bound, sodium-ion pump that catalyses the reversible transfer of methyl groups from tetrahydromethanopterin (H_4_MPT) to CoM, called methyl-H_4_MPT:CoM methyltransferase (Mtr) (Wang et al. 2021, Garcia et al. 2022). Mtr connects the Wood-Ljungdahl pathway (WLP) to Mcr and enables CO_2_-reducing methanogenesis and/or aceticlastic methanogenesis (Fig. 1). Mtr also promotes oxidation of methylated substrates to enable methyl-dismutating methanogenesis. Methanogens that lack Mtr or WLP, must rely on methyl-reducing methanogenesis for their energy metabolism (Wang et al. 2021, Garcia et al. 2022).

### Methyl-dismutating methanogenesis

In the case of methyl-dismutating methanogenesis, which is seen only in *Methanosarcinales*, the methyl groups (Me-X, e.g. methanol, methylated amines, and methylated sulfides) are dismutated to yield methane and CO_2_ in a 3:1 stoichiometry. Twenty five percent of the methyl-groups are oxidized according to Eq. (1) to provide the electrons needed to reduce 75% of the methyl-groups in the form of reduced cofactors such as F_420_H_2_ and reduced ferredoxin (Fd_red_) (Garcia et al. 2022).


(1) \begin{eqnarray*}
{\mathrm{3 MeOH}} + {\mathrm{MeOH}} = 3{\mathrm{ C}}{{\mathrm{H}}_4} + {\mathrm{C}}{{\mathrm{O}}_2} + 2\ {{\mathrm{H}}_2}{\mathrm{O}}.
\end{eqnarray*}

### Methyl-reducing methanogenesis

Methyl-reducing methanogenesis is seen in nature using hydrogen according to Eq. (2), which is referred to in this paper as H_2_-dependent methyl-reducing methanogenesis. In this methanogenesis pathway all methyl groups are reduced to methane (Garcia et al. 2022). Compared to methyl-dismutating methanogenesis presented in Eq. (1), H_2_-dependent methyl-reducing methanogenesis leads to 25% higher methane for the same amount of methylated substrate when using H_2_ as the electron donor compared to metabolism in Eq. (1).


(2) \begin{eqnarray*}
{\mathrm{MeOH}} + {{\mathrm{H}}_2} = {\mathrm{C}}{{\mathrm{H}}_4} + {{\mathrm{H}}_2}{\mathrm{O}}.
\end{eqnarray*}

Hydrogen can be substituted by other electron donors such as formate or ethanol (Hoedt et al. 2016, Steiniger et al. 2022).

*Methanosarcina* species are metabolically versatile, and members of this genus can perform all the major methanogenesis pathways (CO_2_-reducing, aceticlastic, and methyl-dismutating) (Bao et al. 2025). In previous studies, Mtr has been deleted in *Methanosarcina acetivorans and Methanosarcina barkeri*, leading to strains that cannot grow solely on methanol or acetate but require methyl group reduction coupled to acetate oxidation (Welander and Metcalf 2005, Schöne et al. 2022, Bao et al. 2025). In this metabolism, the methyl and the carboxylate group of acetate are oxidized to CO_2_, leading to a 1:4 stoichiometry between acetate and methanol according to Eq. (3), where all methyl groups from methanol are reduced to methane (Fig. 1B).


(3) \begin{eqnarray*}
4{\mathrm{ MeOH}} + {\mathrm{acetat}}{{\mathrm{e}}^ - } + {{\mathrm{H}}^ + } = 4{\mathrm{ C}}{{\mathrm{H}}_4} + 2{\mathrm{ C}}{{\mathrm{O}}_2} + 2\ {{\mathrm{H}}_2}{\mathrm{O}}.
\end{eqnarray*}

*Methanosarcina barkeri* was also shown to be capable of growth using H_2_-dependent methyl-reducing methanogenesis when the methyl-dismutating methanogenesis pathway was disrupted by deleting 5,10-methenyl H_4_MPT cyclohydrolase (Mch). *Methanosarcina acetivorans*, however, is incapable of catabolic H_2_ utilization, which has been hypothesized to be due to its H_2_-independent electron transport chain (Fig. 1A) (Guss et al. 2005).

### Methanosarcina acetivorans

*Methanosarcina acetivorans* is one of the best-studied representatives of the *Methanosarcina* genus and is widely used as a methanogenic model organism due to well-established genetic tools (Metcalf et al. 1997, Pritchett et al. 2004, Nayak and Metcalf 2017, Zhu et al. 2023). When the wildtype strain (*M. acetivorans* C2A) was first discovered, it was able to grow via methyl group dismutation and via aceticlastic methanogenesis, but it could not grow on H_2_ + CO_2_ (Sowers et al. 1984). Trace H_2_ metabolism was reported in *M. acetivorans* with the microbe capable of producing and consuming H_2_ when growing on methanol, acetate and trimethylamine (Lovley and Ferry 1985). Recently, increased methane production has been reported when *M. acetivorans* was grown heterotrophically (i.e. with yeast extract) in presence of H_2_ + CO_2_ and methanol compared to only methanol (Zolkefli et al. 2025). This trace H_2_ metabolism was not enough to support growth, of which is why *M. acetivorans* has not been shown to grow using any H_2_-dependent CO_2_-reducing or H_2_-dependent methyl-reducing methanogenesis pathways yet (Guss et al. 2005). To not rely on H_2_ for electron transport within the cell, which would provide an advantage in environments with competition for hydrogen given the higher threshold of H_2_ in *Methanosarcina* spp., *M. acetivorans* has evolved an H_2_-independent electron transport chain (Feldewert et al. 2020, Zhou et al. 2021).

*Methanosarcina acetivorans* has been shown to grow via CO_2_-reducing methanogenesis using CO as the sole electron donor in a pseudo-wild type strain (Rother and Metcalf 2004). Engineered *M. acetivorans* strains JB-F2 and JB-MF, expressing formate dehydrogenase (Fdh) from *M. barkeri*, are capable of formate-dependent CO_2_-reducing and formate-dependent methyl-reducing methanogenesis, respectively. The formate-dependent methanogenesis pathways of JB-F2 and JB-MF operate in an H_2_-independent manner. Formate-dependent methyl-reducing methanogenesis was made possible in strain JB-MF by disrupting *mtr* (Fig. 1C–I) (Bao et al. 2025).

### Hydrogen and hydrogenases of *M. acetivorans*

The unique feature of *M. acetivorans* is the presence of the *Rhodobacter Nitrogen-Fixation* complex (Rnf) instead of energy-converting hydrogenases (Ech), which allows for an H_2_-independent electron transport chain (Mand and Metcalf 2019, Zhou et al. 2021). Whole genome sequencing of *M. acetivorans*, however, showed the presence of multiple genes encoding hydrogenases such as *frh* (F_420_-reducing hydrogenase), *vht* (initially identified as viologen-reducing hydrogenase two; now known as methanophenazine-reducing hydrogenase), and *vhx* (methanophenazine-reducing hydrogenase), along with a *hyp*-operon, which encodes proteins required for post-translational modification of Vht and Vhx (Galagan et al. 2002, Guss et al. 2009). The genome also encodes genes annotated as *ech-like* or *formate-hydrogen lyase-like* proteins (*hyc-like*) whose protein products we have compared to Ech subunits from *M. barkeri* and Hyc subunits from *E. coli* using NCBI BlastP (Table S1) (McDowall et al. 2014). A hydrogenase deletion study in *M. barkeri* showed that the strain encoding only *fre* and *vhx* had no detectable hydrogenase activity, implying Frh and Vht to be the main hydrogenases for H_2_ utilization (Mand et al. 2018). These main hydrogenases, along with Vhx, have all known active site residues conserved in *M. acetivorans*, suggesting functional expression could make F_420_H_2_ and MPH_2_ available via H_2_ consumption (Fig. 1C–II). Among the promoters, P*vht* is conserved across other Methanosarcina species however P*frh* and P*vhx* are not (Guss et al. 2009). The lack of growth in an H_2_-dependent manner led to the hypothesis that hydrogenases might play some other role besides making electrons available for CO_2_-reduction or methyl-reduction (Galagan et al. 2002). Lack of measurable hydrogenase activity led to the consequent understanding of an H_2_-independent electron transfer chain in *M. acetivorans*, which was hypothesized to be the reason for the robust growth of *M. acetivorans* using CO-dependent CO_2_-reducing methanogenesis compared to that of other H_2_-dependent methanogens such as *Methanothermobacter thermoautotrophicus* and *M. barkeri* (Rother and Metcalf 2004, Guss et al. 2005).

Noteworthy attempts to study H_2_-dependent methyl-reduction in *Methanosarcina* led to the construction of Mch mutants in *M. barkeri* and *M. acetivorans. Methanosarcina barkeri mch1::pac* was capable of H_2_-dependent methyl-reduction, however, in *M. acetivorans*, Mch was essential unless Ech was heterologously expressed. Expression of Ech enabled H_2_-dependent methyl-reduction in *M. acetivorans* with and without disruption of Mch (Guss et al. 2005). Later studies focused on hydrogenase promoter fusions with *uidA* and hydrogenase expression levels on different substrates. *Methanosarcina acetivorans* hydrogenase promoters fused with *uidA* remained inactive in *M. barkeri* as well as *M. acetivorans*, however, *M. barkeri* hydrogenase promoter fusions were expressed in *M. acetivorans* (Guss et al. 2009). Proteomic and microarray experiments had also failed to show expression of *M. acetivorans* hydrogenases above the detection limit (Li et al. 2005a, 2005b). In contrast to the previously mentioned studies, quantitative RT-PCR assays in *M. acetivorans* showed expression of *vht*, suggesting a significant yet unknown role for Vht hydrogenase (Rohlin and Gunsalus 2010). Attempts with inducible promoters hinted at regulation not only at the transcriptional level but also at translational and protein maturation levels (Mand 2018).

Despite multiple efforts, proper conditions to elicit native hydrogenase expression were not identified. A possible insight into the required conditions was hypothesized in a study with connection to nitrogenases in *M. acetivorans*. Diazotrophic growth of *M. acetivorans* showed formation of hydrogen by nitrogenases in addition to fixing nitrogen. It was hypothesized that Vht plays a role in recycling the H_2_ produced during N_2_-fixation (Hoerr et al. 2021, Chanderban and Lessner 2025). The question remains open for other hydrogenases and for the capability of said hydrogenases to participate in catabolism.

### Serine-dependent methyl-reducing methanogenesis

In addition to being a proteogenic amino acid, serine is a one-carbon donor that can donate a formaldehyde equivalent and has therefore been previously studied as a possible methanogenic substrate. Although serine was not a methanogenic substrate, *M. barkeri* cell extracts showed serine hydroxymethyltransferase (SHMT) activity specific to tetrahydrofolate (H_4_F), leading to formation of glycine and methylene-H_4_F (Buchenau and Thauer 2004). This led to the hypothesis of separate folate and pterin pools in *Methanosarcina* that are specific for biosynthetic precursors and methanogenesis, respectively (Lin and Sparling 1995, 1998). However, serine was not studied as a possible reductant in methanogens for methyl-reducing methanogenesis. In a complex, hydrogen-limited environment, from which many methanogens are isolated, knowing if such a novel serine-dependent methyl-reducing methanogenesis pathway exists would add to current knowledge.

Serine-dependent methyl reduction is not yet a known version of the methyl-reducing methanogenesis pathway. Serine can be oxidized via GlyA and FolD, which are present in *M. acetivorans*, to form formyl-H_4_F, which is further incorporated purine metabolism and donate electrons for methyl reduction (Fig. 1C–III).

### Ethanol-dependent methyl-reducing methanogenesis

Ethanol as a reductant for methylotrophic methanogenesis has been reported in *Methanosphaera* sp. WGK-6. *walC* and *walD* gene products were hypothesized to be involved in this pathway (Hoedt et al. 2016). The WalC and WalD protein sequences were used as queries in the NCBI non-redundant protein sequence BLAST database, restricting the taxonomy to *M. acetivorans* C2A, which led to identification of two proteins in *M. acetivorans* C2A (Table S2) annotated as alcohol dehydrogenase and aldehyde dehydrogenase (Fig. 1C—IV). There have been previous reports testing NDMA-dependent alcohol dehydrogenase in *M. barkeri* but no activity was shown with ethanol (Daussmann et al. 1997). The same has not been tested in *M. acetivorans*.

### Our hypothesis and our approach

Given the remarkable flexibility of *M. acetivorans*, we hypothesized that a mutated strain where the *mtr*-operon is disrupted (*Methanosarcina acetivorans* JB-MF), which linked growth and methanogenesis with oxidation of hydrogen and reduction of methanol, could be used to screen electron donors for methyl reduction. This approach had failed previously when Mch was deleted in *M. acetivorans*, but in our approach we aimed to disconnect methanol oxidation at the Mtr level instead of at Mch (Guss et al. 2005). We first tested hydrogen, because it is the most common electron donor for methyl reduction across methanogens. After confirming the viability of hydrogen as an electron donor in *M. acetivorans* JB-MF, we tested candidate hydrogenases Vht and Frh. Deletion of either hydrogenase led to *M. acetivorans* JB-MF being incapable of using hydrogen for methyl-reduction. Finally, we used the strain JB-MF to screen if serine or ethanol could also serve as electron donors for methyl-reduction.

## Materials and methods

### Microbiological methods

*Methanosarcina acetivorans* strains (Table S3) were cultured in a high-salt medium tailored to the specific requirements of the experiment (Sowers et al. 1993). The optical density (OD_600_) of the culture was tracked using a Thermo Spectrophotometer at 600 nm. Five milliliters cultures were grown in Balch tubes with the gas phase (21 ml) consisting of either 72% N_2_/18% CO_2_/10% H_2_S (1% in N_2_) at atmospheric pressure (100 kPa) or 72% H_2_/18% CO_2_/10% H_2_S (1% in N_2_) at atmospheric pressure (100 kPa). Methanol was used as the methylated substrate, with H_2_, serine, or ethanol being tested as the electron donors. Acetate and pyruvate were used as anabolic substrates where specified. Doubling times were calculated using QURVE (Wirth et al. 2023).

### Sampling of cultures for gas analysis and for metabolites in the medium

The first gas samples were taken immediately after inoculation before incubating the cultures at 37°C. Final time point samples were taken at the end of incubation period.

### Methane and hydrogen quantification via GC-FID and GC-TCD

The methane concentrations in the headspace gas samples were measured using a GC-FID (Agilent HP 6890 Gas Chromatograph, Hewlett-Packard), equipped with an HP-PLOT Al_2_O_3_/KCl column (polar alumina phase deactivated with potassium chloride, length, 50 m; diameter, 0.32 mm, thickness, 8 µm). The headspace gas samples (100 µl) were injected with a Gastight 1700 SampleLock Syringe (PN81056, Hamilton). The GC-FID setup comprised the temperature of the front inlet set to 200°C, that of the column oven to 40°C (isothermal), and that of the front detector to 250°C. The pressure of the front inlet was set to 145 kPa, and the total flow of helium was set to 15.2 ml min^−1^. The mobile phase was hydrogen and synthetic air at a flow rate of 35 ml min^−1^ and 350 ml min^−1^, respectively. The makeup flow was 26 ml min^−1^ for helium. A calibration curve was generated using methane standards. Hydrogen was analyzed via gas chromatography with a thermal conductivity detector (GC-TCD 2014, Shimadzu, Japan) equipped with an Agilent J&W packed GC column (1.8 m length, 2 mm Porapak). N_2_ was used as the carrier gas at a flow rate of 20 ml min^−1^. The temperature of the injector, column, and detector was set at 110°C, 80°C, and 110°C, respectively. The volume in the gas bags was measured via a water replacement method, in which the volume of the gas is equal to the volume of the replaced water in the water pillar.

### Metabolite analysis via ^1^H-NMR spectrometry

One milliliter of culture media was sampled with a syringe at the end of growth and centrifuged at 17 000 × g for 120 s. Total of 450 µl of the supernatant was added to an NMR tube, which already contained 50 µl of 1,4-dioxane (1.0% in D_2_O). The tubes were then vigorously shaken and allowed to settle prior to measurements.

The ^1^H-NMR spectroscopy measurements were performed at 25°C on a 400 MHz Bruker Avance III spectrometer (Bruker, Bremen, Germany) equipped with a 5-mm room temperature BBFO probe. Data acquisition was performed using TOPSPIN, version 3.6.2 (Bruker). The spectra were measured using water pre-saturation (32 scans, 12 s relaxation delay, 17 s repetition time). The chemical shifts were calibrated to the signal of dioxane, set to 3.70 ppm. The integration values of signals at 3.30 ppm for methanol (s), 1.57 ppm for acetate (s), 3.88 ppm for serine (dd), and 1.11 ppm for ethanol (t) were used for quantification.

### Strain construction

The process of constructing ∆H_2_ase strains in this study utilized the CRISPR-Cas9 system as previously described (Nayak and Metcalf 2017, Bao et al. 2025). We used the pGGA1 Cas9 vector to introduce gene fragments, each containing ∼1 kb of sequences homologous to regions upstream and downstream of the target genes. These gene fragments were assembled using overlap PCR and the Golden Gate assembly technique (Engler et al. 2008). Detailed information on the gene deletion strains and associated vectors can be found in Table S3. *Methanosarcina acetivorans* JB-MF ∆H_2_ase strains were transformed on media containing methanol (60 mM) + formate (60 mM) + acetate (5 mM) + pyruvate (5 mM) + Casamino acids (1 gl^−1^). Transformation protocols for *Methanosarcina acetivorans* were executed as previously described (Metcalf et al. 1997).

## Results and discussion

### H_2_-dependent growth of the engineered strain *M. acetivorans* JB-MF.

The presence of hydrogen would allow *M. acetivorans*, theoretically, to carry out a combination of methyl-dismutation (Eq. 1) and methyl-reduction using H_2_ (Eq. 2). To ultimately test metabolism in a way that H_2_ is clearly a catabolic substrate in *M. acetivorans* and remove ambiguity between methyl-dismutation or methyl-reduction, we utilized the engineered strain JB-MF, which has Mtr disrupted allowing only for methyl-reduction. We found that when H₂ was used instead of formate, the *mtr* disruption strain remained viable (Table 1), demonstrating that H_2_ can serve as a catabolic electron donor. Electrons for methane formation were derived from H_2_,as no other electron donor was present in the media, and methanol could not be oxidized in strain JB-MF. Viability of the strain suggests a role for the hydrogenases in *M. acetivorans* beyond the previously hypothesized role of recycling nitrogenase-derived hydrogen (Hoerr et al. 2021, Chanderban and Lessner 2025). The expected theoretical yield of methane was 300 µmol, as the targeted initial amount of methanol added to the media was 300 µmol. The measured methane yield was lower than the theoretical expectation because the volatile nature of methanol meant that the targeted methanol concentration in the media was not met. Some hydrogen may also be directed towards anabolic CO_2_-reduction, as *mtr*-disruption meant that cellular carbon could only be sourced from the CO_2_ present in the headspace.

**Table 1:** Methane formation in *M. acetivorans* JB-MF ∆*frh* and ∆*vht* strains.

Strain	Media	Methane (µmol)
JB-MF	MeOH + H_2_	174 ± 26
JB-MF	MeOH + H_2_ + acetate + pyruvate	192 ± 12
JB-MF ∆*vht*	MeOH + H_2_	0.32 ± 0.02
JB-MF ∆*vht*	MeOH + H_2_ + acetate + pyruvate	7 ± 3
JB-MF ∆*frh*	MeOH + H_2_	2.7 ± 0.7
JB-MF ∆*frh*	MeOH + H_2_ + acetate + pyruvate	19.9 ± 0.9

Targeted initial amount: Methanol = 300 µmol, H_2_ = 600 µmol, acetate = 10 µmol, and pyruvate = 10 µmol. Incubation period = 288 h. ± denotes standard deviation (n = 3).

To elucidate which hydrogenase enables growth using H_2_-dependent methyl-reducing methanogenesis in strain JB-MF, we deleted the genes encoding for the hydrogenases Frh and Vht individually, creating two strains labelled JB-MF ∆*frh* and JB-MF ∆*vht*. Neither strain could grow using hydrogen-dependent methyl reduction, as deduced from lack of methane production (Table 1). Supplementing the media with anabolic substrates such as acetate and pyruvate with methanol and H_2_ did not rescue the JB-MF ∆*frh* and JB-MF ∆*vht* strains, implying that the lack of growth was not due to a biosynthetic block. The essentiality of either hydrogenase, despite the presence of the other, for H_2_-dependent methyl-reduction in JB-MF suggests a link between the roles of Frh and Vht hydrogenases that require additional studies. The interplay of Frh and Vht hydrogenase remains unknown even in *M. barkeri* (Mand et al. 2018). H_2_ accumulation was hypothesized as the reason for deletion of Vht being lethal in *M. barkeri* unless Frh was deleted, implying that the cell needs to carefully balance Frh and Vht to maintain redox balance to keep growing (Mand et al. 2018, Mand and Metcalf 2019). JB-MF ∆*frh* and JB-MF ∆*vht* could not grow using H_2_-dependent methyl-reduction, suggesting that this upset in redox balance was lethal even in *M. acetivorans* JB-MF.

### Utilization of strain JB-MF to screen alternative electron donors for methyl-reduction

**Figure 2:**
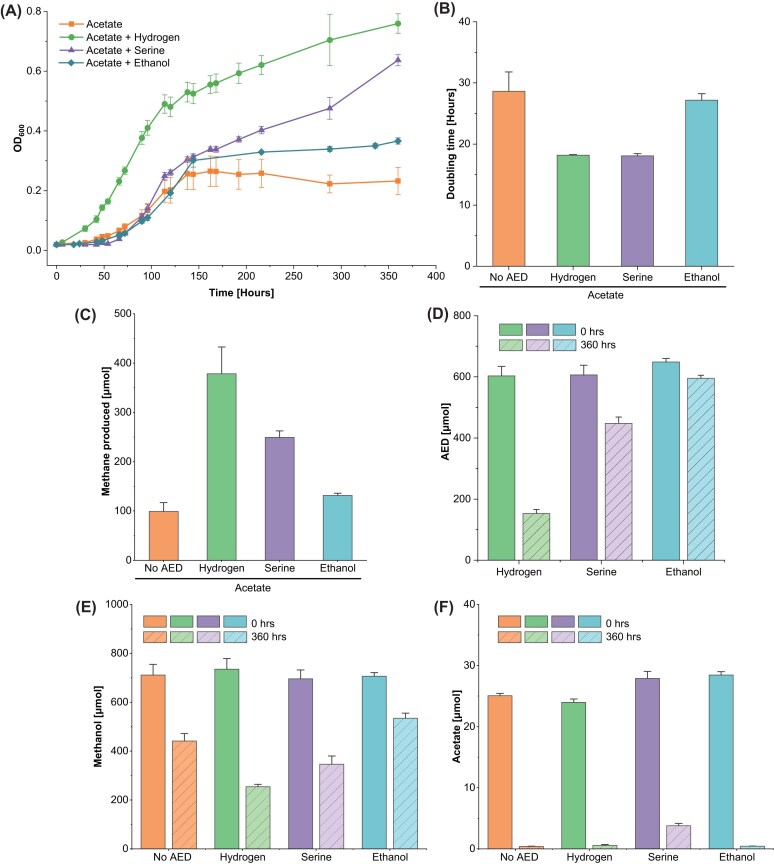
Electron donors- H_2_, serine, ethanol, and no alternative electron donor (No AED) tested for methyl-reducing methanogenesis in *M. acetivorans* JB-MF. (A) OD_600_ (B) Doubling time in hours. (C) Absolute amounts of methane produced in µmol. (D) Residual AED in µmol. (E) Residual methanol in µmol. (F) Residual acetate in µmol. Residual metabolites measured at end of incubation. *X*-axis shows the alternative electron donor. Solid bars show measurement at 0 hrs and striped bars show measurement at 360 hrs. Targeted initial amounts: MeOH = 900 µmol, H_2_ = 600 µmol, serine = 600 µmol, and ethanol = 600 µmol. 30 µmol acetate was provided in all samples as anabolic substrate. Incubation period = 360 h.

Given that strain JB-MF remained viable when formate was replaced with H_2_, we realized that this strain can be used as a chassis to test other native electron donors for methyl-reduction. The strain could also be used as a platform for directed evolution of oxidoreductases, as selection pressure could be applied on these enzymes to provide the necessary reducing equivalents for methyl-reduction in the form of either F_420_H_2_, Fd_red_, or MPH_2_. We tested serine and ethanol as electron donors, comparing them to hydrogen. In addition to methanol (180 mM), we added acetate (6 mM) to avoid any biosynthetic blocks in case the electron donors were not sufficiently reducing to allow CO_2_ fixation. Excess methanol was also provided to allow for calculating electron donor/acceptor ratios with residual electron donor and methanol. Thus, we tested H_2_, serine and ethanol for heterotrophic methyl-reducing methanogenesis in *M. acetivorans* JB-MF (Fig. 2).

“No AED” did not contain any additional electron donor except for the 6 mM acetate. Cultures growing with H_2_-dependent and serine-dependent methyl-reducing methanogenesis had significantly lower doubling time and significantly higher OD_600_ and methane production (One-way ANOVA, *P* < 0.001; Fig. 2). The methane produced in the acetate only sample matches the metabolism provided in Eq. (3), with ∼25 µmol acetate leading to ∼100 µmol methane. Similarly, adjusting for methane due to electrons from acetate for samples with hydrogen and serine show every eight molecules of hydrogen and serine leads to formation of five and seven molecules of methane, respectively (Table S4). Although cultures with ethanol had significantly higher OD_600_, ethanol did not lead to measurable increase in methane production or decrease in doubling time (One-way ANOVA, *P* < 0.05 for OD_600_; Fig. 2). No ethane was detected in the cultures supplemented with ethanol as ethane production starts after all methanol is consumed in the media (Fig. 2) (Belay and Daniels 1988, Somvanshi et al. 2025).

We analyzed the cultures at the end of growth to confirm consumption of the provided electron donors. All cultures had consumed acetate, as expected (Fig. 2). The measured consumption of methanol is higher than the measured methane formation, possibly due to the volatile nature of methanol that would cause methanol to be in the vapor phase in the Balch tubes and would lead to lower estimation of methanol in cultures (Fig. 2B and E). All electron donors were significantly consumed by *M. acetivorans* (Two-way ANOVA, H_2_ and serine: *P* < 0.0001, Ethanol: *P* < 0.05; Fig. 2D). It is possible that the consumed ethanol contributes to higher final OD_600_ but did not lead to increased methane production within the timeframe measured. Much longer incubation periods could have led to accumulation of trace methane, which could have been seen as significant increase in methane production. The serine oxidation pathway in *M. acetivorans* leads to purine metabolism via formyl-H_4_F. Heterologous expression of formate-tetrahydrofolate ligase (*fhs*) in *M. acetivorans* JB-MF would lead to a strain where serine is completely oxidized to CO_2_, yielding an additional ATP and F_420_H_2_ in the process. As serine would be an integral part of the energy metabolism of *M. acetivorans* JB-MF in absence of other electron donors, it provides deletion of GlyA as a possibility for constructing serine auxotrophic mutant.

## Conclusion

Disrupting the methyl-dismutating pathway at Mtr (strain JB-MF) enabled consistent consumption of hydrogen as a catabolic electron donor. Vht and Frh were found to be essential in JB-MF, as JB-MF ∆*frh* and JB-MF ∆*vht* could not grow on methanol and hydrogen, even in the presence of anabolic substrates, acetate and pyruvate. The plasticity of energy metabolism in *M. acetivorans* enabled us to use strain JB-MF to probe for different electron donors for methyl reduction and revealed that hydrogen and serine can act as alternative electron donors for methyl-reducing methanogenesis. Further studies would elucidate the exact path electrons take from hydrogen and serine to enable methyl-reduction. The regulation of hydrogenases in *M. acetivorans* is still poorly understood, however, the microbe does encode functional hydrogenases, allowing for hydrogen consumption. Our study shows that the metabolic flexibility of *M. acetivorans* is still underestimated and extends to hydrogen-dependent and serine-dependent methyl-reducing methanogenesis, of which the latter is a novel methanogenic pathway.

## Supplementary Material

fnag061_Supplemental_Files

## Data Availability

All study data and corresponding statistical analyses are available in the published article and/or the Supplementary material.

## References

[bib1] Bao J, Somvanshi T, Tian Y et al. Nature AND nurture: enabling formate-dependent growth in *Methanosarcina acetivorans*. FEBS J. 2025;292:225171. 10.1111/febs.17409. 39887878

[bib2] Belay N, Daniels L. Ethane production by Methanosarcina barkeri during growth in ethanol supplemented medium. Antonie Van Leeuwenhoek. 1988;54:113–25. 10.1007/BF00419199. 3395108

[bib3] Buchenau B, Thauer RK. Tetrahydrofolate-specific enzymes in *Methanosarcina barkeri* and growth dependence of this methanogenic archaeon on folic acid or p-aminobenzoic acid. Arch Microbiol. 2004;182:31325. 10.1007/s00203-004-0714-0. 15349715

[bib4] Chanderban M, Lessner DJ. ModE regulates alternative nitrogenase expression in the methanogen *Methanosarcina acetivorans*. Mol Microbiol. 2025;124:14. 10.1111/mmi.15377. PMC12327845 40353477

[bib5] Daussmann T, Aivasidis A, Wandrey C. Purification and characterization of an alcohol: N,N -dimethyl-4-nitrosoaniline oxidoreductase from the methanogen *Methanosarcina Barkeri* DSM 804 strain fusaro. Eur J Biochem. 1997;248:88996. 10.1111/j.1432-1033.1997.00889.x. 9342243

[bib6] Engler C, Kandzia R, Marillonnet S. A one pot, one step, precision cloning method with high throughput capability. PLoS One. 2008;3:e3647. 10.1371/journal.pone.0003647. 18985154 PMC2574415

[bib7] Feldewert C, Lang K, Brune A. The hydrogen threshold of obligately methyl-reducing methanogens. FEMS Microbiol Lett. 2020;367:fnaa137. 10.1093/femsle/fnaa137. 32821944 PMC7485788

[bib8] Galagan JE, Nusbaum C, Roy A et al. The genome of *M. acetivorans* reveals extensive metabolic and physiological diversity. Genome Res. 2002;12:53242. 10.1101/gr.223902. PMC187521 11932238

[bib9] Garcia PS, Gribaldo S, Borrel G. Diversity and evolution of methane-related pathways in archaea. Annu Rev Microbiol. 2022;76:72755. 10.1146/annurev-micro-041020-024935. 35759872

[bib10] Guss AM, Kulkarni G, Metcalf WW. Differences in hydrogenase gene expression between *Methanosarcina acetivorans* and *Methanosarcina barkeri*. J Bacteriol. 2009;191:282633. 10.1128/JB.00563-08. PMC2668380 19201801

[bib11] Guss AM, Mukhopadhyay B, Zhang JK et al. Genetic analysis of *mch* mutants in two *Methanosarcina* species demonstrates multiple roles for the methanopterin-dependent C-1 oxidation/reduction pathway and differences in H2 metabolism between closely related species. Mol Microbiol. 2005;55:167180. 10.1111/j.1365-2958.2005.04514.x. 15752192

[bib12] Hoedt EC, Cuív PÓ, Evans PN et al. Differences down-under: alcohol-fueled methanogenesis by archaea present in Australian macropodids. ISME J. 2016;10:237688. 10.1038/ismej.2016.41. PMC5030694 27022996

[bib13] Hoerr JM, Dhamad AE, Deere TM et al. Vht hydrogenase is required for hydrogen cycling during nitrogen fixation by the non-hydrogenotrophic methanogen *Methanosarcina acetivorans*. Biorxiv. 2021. 10.1101/2021.10.12.464174.

[bib14] Li Q, Li L, Rejtar T et al. Proteome of *Methanosarcina acetivorans* part I: an expanded view of the biology of the cell. J Proteome Res. 2005a;4:11228. 10.1021/pr049832c. 15707366

[bib15] Li Q, Li L, Rejtar T et al. Proteome of *Methanosarcina acetivorans* part II: comparison of protein levels in acetate- and methanol-grown cells. J Proteome Res. 2005b;4:12935. 10.1021/pr049831k. 15707367

[bib16] Lin Z, Sparling R. Oxidation–reduction of methanol, formaldehyde, serine, and formate in *Methanosphaera stadtmanae* using ^14^C short- and long-term labelling. Can J Microbiol. 1995;41:104853. 10.1139/m95-146.

[bib17] Lin Z, Sparling R. Investigation of serine hydroxymethyltransferase in methanogens. Can J Microbiol. 1998;44:6526. 10.1139/w98-050. 9783425

[bib18] Lovley DR, Ferry JG. Production and consumption of H_2_ during growth of *Methanosarcina* spp. on acetate. Appl Environ Microb. 1985;49:2479. 10.1128/aem.49.1.247-249.1985. PMC238382 16346703

[bib19] Mand TD, Kulkarni G, Metcalf WW. Genetic, biochemical, and molecular characterization of *Methanosarcina barkeri* mutants lacking three distinct classes of hydrogenase. J Bacteriol. 2018;200:e00342–18. 10.1128/JB.00342-18. 30012731 PMC6153667

[bib20] Mand TD, Metcalf WW. Energy conservation and hydrogenase function in methanogenic archaea, in particular the genus *Methanosarcina*. Microbiol Mol Biol Rev. 2019;83:e00020–19. 10.1128/MMBR.00020-19. 31533962 PMC6759668

[bib21] Mand TD. Hydrogenase utilization and regulation in species of *Methanosarcina*. 2018.

[bib22] McDowall JS, Murphy BJ, Haumann M et al. Bacterial formate hydrogenlyase complex. Proc Natl Acad Sci USA. 2014;111:E3948–56. 10.1073/pnas.1407927111. 25157147 PMC4183296

[bib23] Metcalf WW, Zhang JK, Apolinario E et al. A genetic system for Archaea of the genus *Methanosarcina *: liposome-mediated transformation and construction of shuttle vectors. Proc Natl Acad Sci USA. 1997;94:262631. 10.1073/pnas.94.6.2626. PMC20139 9122246

[bib24] Nayak DD, Metcalf WW. Cas9-mediated genome editing in the methanogenic archaeon *Methanosarcina acetivorans*. Proc Natl Acad Sci USA. 2017;114:297681. 10.1073/pnas.1618596114. PMC5358397 28265068

[bib25] Pritchett MA, Zhang JK, Metcalf WW. Development of a markerless genetic exchange method for *Methanosarcina acetivorans* C2A and its use in construction of new genetic tools for methanogenic archaea. Appl Environ Microb. 2004;70:142533. 10.1128/AEM.70.3.1425-1433.2004. PMC368415 15006762

[bib26] Rohlin L, Gunsalus RP. Carbon-dependent control of electron transfer and central carbon pathway genes for methane biosynthesis in the Archaean, *Methanosarcina acetivorans* strain C2A. BMC Microbiol. 2010;10:62. 10.1186/1471-2180-10-62. 20178638 PMC2838876

[bib27] Rother M, Metcalf WW. Anaerobic growth of *Methanosarcina acetivorans* C2A on carbon monoxide: an unusual way of life for a methanogenic archaeon. Proc Natl Acad Sci USA. 2004;101:1692934. 10.1073/pnas.0407486101. PMC529327 15550538

[bib28] Schöne C, Poehlein A, Jehmlich N et al. Deconstructing *Methanosarcina acetivorans* into an acetogenic archaeon. Proc Natl Acad Sci USA. 2022;119:e2113853119. 34992140 10.1073/pnas.2113853119 PMC8764690

[bib29] Somvanshi T, Tran MA, Bao J et al. Methanosarcina acetivorans requires methanol:coenzyme M methyltransferases for ethane formation from ethanol. Antonie van Leeuwenhoek. 2025;118:154. 10.1007/s10482-025-02165-x. 40974455 PMC12450230

[bib30] Sowers KR, Baron SF, Ferry JG. *Methanosarcina acetivorans* sp. nov., an acetotrophic methane-producing bacterium isolated from marine sediments. Appl Environ Microb. 1984;47:9718. 10.1128/aem.47.5.971-978.1984. PMC240030 16346552

[bib31] Sowers KR, Boone JE, Gunsalus RP. Disaggregation of *Methanosarcina* spp. and growth as single cells at elevated osmolarity. Appl Environ Microb. 1993;59:38329. 10.1128/aem.59.11.3832-3839.1993. PMC182538 16349092

[bib32] Steiniger F, Sorokin DY, Deppenmeier U. Process of energy conservation in the extremely haloalkaliphilic methyl-reducing methanogen *Methanonatronarchaeum thermophilum*. FEBS J. 2022;289:54963. 10.1111/febs.16165. 34435454

[bib33] Thauer RK, Kaster A-K, Seedorf H et al. Methanogenic archaea: ecologically relevant differences in energy conservation. Nat Rev Micro. 2008;6:57991. 10.1038/nrmicro1931. 18587410

[bib34] Wang Y, Wegener G, Williams TA et al. A methylotrophic origin of methanogenesis and early divergence of anaerobic multicarbon alkane metabolism. Sci Adv. 2021;7:eabj1453. 10.1126/sciadv.abj1453. 34215592 PMC11057702

[bib35] Welander PV, Metcalf WW. Loss of the *mtr* operon in *Methanosarcina* blocks growth on methanol, but not methanogenesis, and reveals an unknown methanogenic pathway. Proc Natl Acad Sci USA. 2005;102:106649. 10.1073/pnas.0502623102. PMC1180775 16024727

[bib36] Wirth NT, Funk J, Donati S et al. QurvE: user-friendly software for the analysis of biological growth and fluorescence data. Nat Protoc. 2023;18:24013. 10.1038/s41596-023-00850-7. 37380826

[bib37] Zhou J, Holmes DE, Tang H-Y et al. Correlation of key physiological properties of *Methanosarcina* isolates with environment of origin. Appl Environ Microb. 2021;87:e00731–21. 10.1128/AEM.00731-21. PMC8316034 33931421

[bib38] Zhu P, Somvanshi T, Bao J et al. CRISPR/Cas12a toolbox for genome editing in *Methanosarcina acetivorans*. Front Microbiol. 2023;14:1235616. 10.3389/fmicb.2023.1235616. 38149272 PMC10750270

[bib39] Zolkefli N, Shui X, Ma K et al. Unveiling the impact of indole derivatives on methanogenic archaea and microbial functions in anaerobic digestion of waste sewage sludge. Appl Biochem Biotechnol. 2025;198:99. 10.1007/s12010-025-05387-x. 41205042

