# Supplementary material for: Disrupting the mtr-operon in Methanosarcina acetivorans enables methyl-reducing methanogenesis with hydrogen and serine as the alternative electron donors
Source: FEMS Microbiol Lett. 2026 May 22;373:fnag061. doi: 10.1093/femsle/fnag061 (PMC13224831; doi:10.1093/femsle/fnag061)
Supplement: fnag061_Supplemental_Files [file fnag061_supplemental_files.zip › Revised Supplementary Information.docx]

**Supplementary information-**

**SI Tables**

**Table S1. BlastP results for ethanol oxidation hypothesis presented in Fig 1C**

| **Gene number** | **Gene product** | **Aligned to** | **Query Cover** | **Identities** | **Positives** | **E value** |
| --- | --- | --- | --- | --- | --- | --- |
| MA_2630 | Alcohol dehydrogenase | WalC  (*Methanosphaera* sp WGK-6) | 87% | 37.85% | 56% | 1e-72 |
| MA_0705 | Aldehyde dehydrogenase | WalD  (*Methanosphaera* sp WGK-6) | 96% | 28.08% | 44% | 1e-54 |

**Table S2. BlastP results for Ech-like proteins in *M. acetivorans***

| **Gene number** | **Gene Product** | **Aligned to** | **Query Cover** | **Identities** | **Positives** | **E value** |
| --- | --- | --- | --- | --- | --- | --- |
| MA_4371 | echA-like | echA (*M. barkeri*) | 73% | 27.60% | 47% | 9e-30 |
| MA_4369 | echB-like | echB (*M. barkeri*) | 74% | 26.58% | 48% | 1e-16 |
| MA_4373 | HycG-like | echC (*M. barkeri*) | 70% | 42.02% | 61% | 2e-35 |
| MA_4373 | HycG-like | HycG (*E. coli*) | 54% | 42.55% | 56% | 3e-37 |
| MA_4372 | HycE-like | echD (*M. barkeri*) | 18% | 26.04% | 47% | 4e-07 |
| MA_4372 | HycE-like | HycE (*E. coli*) | 82% | 32.15% | 50% | 5e-78 |
| MA_4372 | HycE-like | echE (*M. barkeri*) | 67% | 23.31% | 44% | 7e-32 |
| MA_4370 | 4Fe-4S ferredoxin-type domain-containing protein | echF (*M. barkeri*) | 22% | 32.84% | 55% | 2e-11 |
| MA_4368 | HycC-like | HycC (*E. coli*) | 74% | 34.93% | 52% | 1e-67 |

**Table S3. Deletion strains and plasmid maps used in this study.**

| Strain name | Genotype | Source | Vector name | Vector maps |
| --- | --- | --- | --- | --- |
| JB-MF | WWM73 ΔmtrED(1-44)::fdh-Tfpo_Fusaro | (Bao *et al.* 2025) | |  |
| JB-MF ∆*frh* | JB-MF ∆frhADGB(1-851) | This study | pGGA Frh | <https://benchling.com/s/seq-KBHYUchHkpOVh6ku9dX6?m=slm-7w4HmsihDtOFEAcWJUvl> |
| JB-MF ∆*vht* | JB-MF ∆vhtG(591-1152)ACD-TvhtD(1-311) | This study | pGGA vht | <https://benchling.com/s/seq-t0w4p8wNFMpoomTiJIqq?m=slm-RYfQchN98Kk6j3glkgPh> |

**Table S4. Ratio of methane produced to AED consumed**

| **AED** | **Average CH_4_ in µmol** | **Average acetate at T0 in µmol** | **Average methane from acetate in µmol** | **Average methane from AED in µmol** | **AED consumption in µmol** | **CH4/AED ratio** | **Nearest fraction** |
| --- | --- | --- | --- | --- | --- | --- | --- |
| **H_2_** | 378.36 | 23.97 | 95.88 | 282.48 | 450.37 | 0.63 | 5/8 |
| **Serine** | 249.29 | 27.88 | 111.52 | 137.77 | 158.66 | 0.87 | 7/8 |
| **Ethanol** | 131.69 | 28.45 | 113.8 | 17.89 | 53.15 | 0.34 | 1/3 |

**References**

Bao J, Somvanshi T, Tian Y *et al.* Nature AND nurture: enabling formate-dependent growth in Methanosarcina acetivorans. *FEBS J* 2025;**292**:2251–71.
